# Directions for Research on Climate and Conflict

**DOI:** 10.1029/2020EF001532

**Published:** 2020-07-03

**Authors:** Katharine J. Mach, W. Neil Adger, Halvard Buhaug, Marshall Burke, James D. Fearon, Christopher B. Field, Cullen S. Hendrix, Caroline M. Kraan, Jean‐Francois Maystadt, John O'Loughlin, Philip Roessler, Jürgen Scheffran, Kenneth A. Schultz, Nina von Uexkull

**Affiliations:** ^1^ Rosenstiel School of Marine and Atmospheric Science University of Miami Miami FL USA; ^2^ Leonard and Jayne Abess Center for Ecosystem Science and Policy University of Miami Coral Gables FL USA; ^3^ Geography, College of Life and Environmental Sciences University of Exeter Exeter UK; ^4^ Peace Research Institute Oslo Oslo Norway; ^5^ Department of Sociology and Political Science Norwegian University of Science and Technology Trondheim Norway; ^6^ Department of Earth System Science Stanford University Stanford CA USA; ^7^ National Bureau of Economic Research Cambridge MA USA; ^8^ Department of Political Science Stanford University Stanford CA USA; ^9^ Stanford Woods Institute for the Environment Stanford University Stanford CA USA; ^10^ Korbel School of International Studies University of Denver Denver CO USA; ^11^ Peterson Institute for International Economics Washington DC USA; ^12^ Environmental Science and Policy Graduate Program, Leonard and Jayne Abess Center for Ecosystem Science and Policy University of Miami Coral Gables FL USA; ^13^ Institute of Development Policy (IOB) University of Antwerp Antwerp Belgium; ^14^ Department of Economics Lancaster University Lancaster UK; ^15^ Institute of Behavioral Science and Department of Geography University of Colorado Boulder Boulder CO USA; ^16^ Department of Government College of William & Mary Williamsburg VA USA; ^17^ Research Group Climate Change and Security (CLISEC), Institute of Geography University of Hamburg Hamburg Germany; ^18^ Department of Peace and Conflict Research Uppsala University Uppsala Sweden

**Keywords:** climate change, armed conflict

## Abstract

The potential links between climate and conflict are well studied, yet disagreement about the specific mechanisms and their significance for societies persists. Here, we build on assessment of the relationship between climate and organized armed conflict to define crosscutting priorities for future directions of research. They include (1) deepening insight into climate‐conflict linkages and conditions under which they manifest, (2) ambitiously integrating research designs, (3) systematically exploring future risks and response options, responsive to ongoing decision‐making, and (4) evaluating the effectiveness of interventions to manage climate‐conflict links. The implications of this expanding scientific domain unfold in real time.

## Key Points

- Disagreement about the relationship between climate and the risk of violent conflict persists, limiting societal management of the risks
- Future directions for climate‐conflict research include deepening insight on what the links are, when they matter, and how they manifest
- Compelling opportunities necessitate integrating research designs, responsive to ongoing decision‐making and potential responses

## 1 Introduction

The potential links between climate and the risk of violent conflict are well studied, with agreement that the issue raises concerns about the scope and severity of possible climate change impacts (Adger et al., 2014; Burke et al., 2015). Yet the scholarship has yielded divergent findings and controversy about the direct and identifiable causal mechanisms and their significance relative to other sources of conflict.

We are a group of scholars from different disciplines, including environmental science, political science, geography, and economics, who have reached varying conclusions in our previous work on climate and conflict. Together, we assessed the current state of knowledge on the relationship between climate and organized armed conflict within countries (Mach et al., 2019a). Our expert assessment combined full‐day individual elicitation interviews and a subsequent 2‐day group deliberation and yielded 950 transcript pages reflecting on areas of agreement and the reasons for disagreement. Here, we build on that assessment to define crosscutting priorities for future directions of research within a broad and expanding scholarly domain.

Across the expert group, best estimates were that 3–20% of organized armed conflict risk has been influenced by climate over the last century (Mach et al., 2019a). Other drivers, however, were judged much more influential for conflict overall, and the specific mechanisms underpinning climate‐conflict linkages were identified as a key uncertainty. We agreed that additional climatic changes, including changes in climate extremes and resulting impacts for societies and economies, will further increase the risks of civil conflict although uncertainties grow larger.

**Figure 1:**
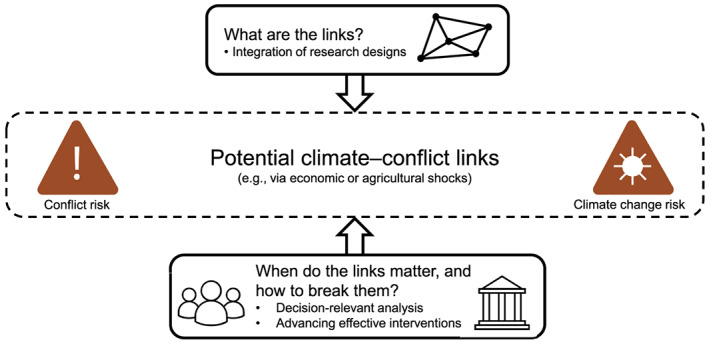
Directions for research on climate and conflict. Future research has compelling opportunities to deepen insight on potential climate‐conflict links. This work can be responsive to the needs for ongoing decision‐making and responses.

Determining the relative importance of climate versus other drivers of conflict remains a key starting point for future research (Mach et al., 2019a). This need includes the extent to which climate‐conflict linkages are moderated by political and socioeconomic conditions that could lead these impacts to change over time (Gleditsch, 2012; Koubi, 2019). The links will play out differently across scales, from individual‐level violence, to civil war, to interstate conflict. Here we recognize that much research initially explored climate‐conflict links for organized armed conflict within countries (e.g., Adger et al., 2014), as reflected in our treatment, at the same time that the path forward requires attention to the diverse forms of social contestation and violence. New and better models and decision‐support methods are also necessary to enrich knowledge of the uncertainties that will persist and must be managed as such. Climate change impacts continue to intensify, reductions of heat‐trapping emissions remain ineffective at global scale, and choices to act on potential climate‐conflict linkages or not—whichever may be more prudent—carry implications that unfold in real time. In the sections that follow, we define crosscutting priorities for future directions of integrative, decision‐relevant research toward these themes (Figure 1).

## 2 Deeper Insight Into Climate‐Conflict Linkages

Research to date has identified some statistical signals of climatic effects on conflict. The challenge is to go beyond black‐box approaches and address the mechanisms linking climate and armed conflict, the conditions under which they materialize, and the relative contribution of climate among conflict determinants. This challenge (Buhaug, 2015; Gemenne et al., 2014; Gleditsch, 2012; Selby, 2014) is especially important for informing interventions and management of the risks from the onset and escalation of violence through to its termination. The links are also integral to human security impacts well short of violence, which will disproportionately affect vulnerable communities. Hurdles include the diversity of possibly relevant mechanisms across different settings and the difficulty of collecting data in conflict‐prone regions (Hendrix, 2017; Salehyan, 2014).

Most plausible explanations work through economic shocks related to climate (Mach et al., 2019a). Such shocks, in turn, are thought to decrease the opportunity costs of participating in rebellion, exacerbate inequality and disrupt cooperative bargains among groups, and hamper long‐term socioeconomic development. To the extent that these mechanisms lead to conflict through observable intermediate steps, one option is to trace the relevant processes, starting with conflicts influential in statistical results and evaluating whether they arose through hypothesized mechanisms (Lyall, 2015). Mixed methods can then iterate between in‐depth qualitative study of specific cases and crosscutting quantitative analysis.

For example, process tracing methods could focus on the opportunity‐cost mechanism in which climate‐related hazards adversely impact livelihoods, such as through reducing agricultural income, and thereby make participation in violence relatively more attractive (Koubi, 2019; Maystadt & Ecker, 2014). Market reactions, such as commodity price shocks, may simultaneously play a role (Dube & Vargas, 2013; McGuirk & Burke, 2017). There are also likely to be complex interactions between resource scarcity and abundance (Koren, 2019). Process tracing could additionally assess whether and how uneven economic impacts disrupt cooperative interactions among groups. Consideration of differential effects across societal groups and locations is important. Studying cases experiencing shocks, such as widespread failure of export crops, but not violence is crucial for understanding the moderators of outcomes for societies (Gemenne et al., 2014).

Monitoring contexts with climatic shocks, including through multicountry, multisite panel surveys in drought‐prone regions, can shed light on operational mechanisms, for instance, the ways livelihood choices or adaptive adjustments shape attitudes toward violence and participation in conflict (Koren, 2019; Linke et al., 2017; Vestby, 2019). Evaluation can encompass microlevel effects, such as through indicators of willingness to cooperate with people from other societal groups or through household‐level capital accumulation and well‐being (Pande & Savenije, 2016).

There are also opportunities to incorporate the burgeoning field of single‐event attribution, in which the role of human‐induced climate change can be probabilistically assessed in individual heat, rainfall, or drought events (Knutson et al., 2017; Otto, 2017). These climate‐science advances could be used to bridge the long‐standing disconnect between weather and climate in the analysis and interpretation of consequences for conflict (Buhaug, 2015).

Randomized controlled trials or quasi‐experimental evaluation of existing programs could both help adjudicate among alternative mechanisms and inform understanding of the effectiveness of particular policy interventions (Fetzer, 2020). For example, such studies could evaluate whether recipients of cash transfers or crop insurance are less likely to engage in violence compared to control groups not receiving such support (e.g., Buller et al., 2018; Crost et al., 2016). They could also assess the implications of larger‐scale interventions, such as infrastructural interventions to support water security or mitigate flood risk, which may displace marginalized populations and lead to resistance and unrest (e.g., Mills‐Novoa & Taboada Hermoza, 2017). Governance challenges related to equity, power, and justice are central in associated policies (Srinivasan et al., 2012; Zwarteveen & Boelens, 2014).

## 3 Integration Across Research Designs

Divergent research methods have resulted in different findings about the relationship between climate variability and conflict across both quantitative and qualitative studies (Buhaug et al., 2014; Hsiang et al., 2013; Selby, 2014). There have been different conclusions about the same episodes of conflict arising from differing applications of similar research methods (e.g., Burke et al., 2015), as well as from fundamental differences across modes of inquiry and epistemologies (e.g., crosscutting statistical analyses testing hypotheses versus in‐depth studies generating theories; Selby, 2014).

While such differences contribute to a lack of consensus, they also create an opportunity. Systematic evaluation of the sensitivity of results to different research designs and data choices would explain the implications of the conclusions that arise under some data, modeling, and analysis choices but not others. When do they suggest artifacts versus relationships that depend on the social context? What is being missed when key potential drivers of engagement in violence cannot be quantified or are unknown?

Increasingly, scholarship has recognized the need to move beyond large‐scale, continent wide analyses to within‐country multidisciplinary evaluations in order to understand climate‐conflict mechanisms. The range of research designs to integrate includes, importantly, qualitative inquiry such as through ethnographic participant observations and semistructured interviews (Ide, 2017; LeBillon & Duffy, 2018). A trade‐off to contend with, however, is the potential nonrepresentativeness of the places that can be readily studied. For example, scholarship on climate and conflict has focused on particular regions or high‐profile contexts, such as Kenya or Syria, because of the accessibility of data, feasibility of research, or scope and prominence of the example (Hendrix, 2017; Selby, 2014; Selby et al., 2017).

Future research can exploit growing access to microlevel data from diverse sources, such as satellite and drone imagery, social media, and population surveys. These data sources shed light on diverse factors from fine‐scale differences in agricultural productivity, to the movement and interactions of people, to levels of trust in government (e.g., Ash & Obradovich, 2019). Different methods can be applied to such data sets. Machine learning and other data‐science methods that flexibly accommodate complex and conditional relationships can be used to anticipate dynamics of conflict where data on violence are sufficient (e.g., Hegre et al., 2019; Mueller & Rauh, 2018). Microlevel data can also advance the measurement of potential climate‐conflict pathways previously only qualitatively or coarsely understood. For example, data sets on economic well‐being or asset wealth at the household or village scale could enable subsequent analysis of their role for climate and conflict (Jean et al., 2016). Tracking the mobility of people through cellphone or social media data or repeated panel surveys can shed light on migratory mechanisms and implications for the stability of societies (Lu et al., 2016).

## 4 Decision‐Relevant Exploration of Future Risks and Options

Analyzing future conflict risk is inherently difficult whether in the context of early warning or long‐term scenario‐based projections (Hegre et al., 2016; Witmer et al., 2017). Existing models are better at predicting which states or regions are more vulnerable to conflict over the long term than they are at predicting whether violence will break out in a given place at a given time or if and when it will escalate. This, in turn, is a challenge for proactive intervention, peacekeeping missions, development aid, or food assistance. Forecasting has to grapple with intrinsically unpredictable strategic adjustments by government and members of society as information emerges. At larger scales, anticipation of conflict risk faces other issues of out‐of‐sample prediction (Bowlsby et al., 2019; Buhaug & Vestby, 2019; Van Weezel, 2019; Walter, 2017). Identified relationships between climatic variability and increased instability may well hold over a given window of time or set of contexts, yet fail beyond that (Abel et al., 2019). It is important to understand such dynamics to shed light on the generalizability versus context specificity of different theories and results.

Evaluating possible futures to inform decision‐making requires exploring local‐to‐global interactions relevant to both climate change and conflict risk. If climate‐related shocks affect flows of displaced people or the functioning of agricultural markets, conflict could arise in times and places that are distant from the actual trigger. For example, a combination of weather shocks and export bans in major grain‐producing countries led global food prices to spike in 2010, with destabilizing effects for importers in the Middle East and North Africa (Costello et al., 2015). Further, cultural values underpin what is at stake, such as for natural resources that have place‐specific meanings or histories distinct from their economic value. Iterative interactions between analysts and practitioners are therefore central in advancing modeling capacity and iteratively informing decision‐maker goals and priorities.

There are also uncertainties unlikely to be eliminated or even reduced. Some system interactions are unknowably complex and the drivers, such as geopolitical factors shaping external power interventions, difficult to anticipate (Mach et al., 2019a). Such deep uncertainties include the extent and effectiveness of adaptation, lack of historical precedents, and the tail risks of low‐probability outcomes. A range of analytical tools are relevant including scenario simulations, preferences elicitation, agent‐based modeling, dynamic adaptive pathways, or theories of tipping points, risk cascades, and stability (Hegre et al., 2019; Kwakkel et al., 2016; Witmer et al., 2017). Nuanced risk communication and policy analyses robust to the uncertainties are essential.

## 5 Interventions That Work

Ultimately, the most consequential open questions relate to the effectiveness of interventions that might break the links between climate change, armed conflict, and instability, as emphasized by practitioners managing these risks in intergovernmental, national, and local contexts (Busby, 2018; Mach et al., 2019b). An important question is whether standard techniques of conflict management (e.g., mediation, peacekeeping, and aid) are sufficient or if new interventions at interacting scales are needed to manage human security and conflict risks arising from climate change. The associated interconnections stretch from global‐scale cooperation on greenhouse gas emissions reductions, through to village‐scale interventions to manage the impacts. Some toolkits, such as transboundary institutions for water management, are better understood than others (e.g., within‐country institutions for managing conflict risks in resource‐scarce regions) (Busby, 2018).

Policy‐focused analyses could evaluate specific interventions related to adaptation, insurance and risk sharing, humanitarian assistance, or development policy, considering implications for conflict risk (Di Falco et al., 2014; Fetzer, 2020). Which policies and measures addressing climate‐related economic shocks, reduced agricultural production, or displacement reduce engagement in violence? Experimental field methods are unlikely to examine the full link between climate and conflict, yet can instead explore intermediary outcomes predicted by different mechanisms, such as increased mistrust of government following inadequate and unequal distribution of disaster relief (e.g., Humphreys & Weinstein, 2009). Conducting such experiments, and translating their findings into improved policies, necessitates sophisticated attention to the ethics of research potentially posing risks for people studied (Israel & Hay, 2011). It also demands dedication from researchers and practitioners alike.

Fundamental to interventions that work is understanding what enables cooperation and peace in conflict and postconflict situations and in circumstances of climate‐related shocks, as well as the ways in which conflict and cooperation coexist (Gemenne et al., 2014; Ide & Scheffran, 2014; Zeitoun & Mirumachi, 2008). Relevant interventions pertain to the integration of displaced populations and social‐safety‐net support to most marginalized groups as well as natural resource management and environmental cooperation as a facilitator for environmental peacebuilding (Ide, 2018). There is also an associated shift in framing, toward the capabilities of people and government institutions and their security more holistically (Linke et al., 2017).

As climate change impacts and responses extend beyond historical experiences, new questions will arise. For migration, for example, if governments increasingly relocate populations or face issues with refugee resettlement, testing implementation processes to minimize conflict will have immediate relevance. Or if involuntary displacement intensifies, there will be increased urgency in evaluating approaches and conditions under which displaced persons are effectively absorbed into communities in ways that minimize frictions, within countries and in countries both bordering and far removed (Linke et al., 2018; Maystadt et al., 2019).

Coproduction of research among researchers, stakeholders, practitioners, or boundary organizations is potentially fruitful (Busby, 2018; Lemos et al., 2018). Such collaborations encourage real‐world orientations on how best to proactively address potential linkages between climate and conflict across contexts or on how to implement adaptation in fragile states or conflict situations (Abrahams, 2020). International peacekeeping groups, development banks, and disaster relief organizations are all working in contexts where climate‐conflict links are potentially relevant, and they could be informed by evidence on interactions between their investments and such links. As climate change adaptation increasingly occurs, evaluation of interventions implemented by different organizations and agencies could additionally yield insights toward improving their conflict sensitivity (e.g., Benjaminsen & Bryceson, 2012). The considerations importantly include the risks of downplaying versus overemphasizing conflict risks from climate change given current knowledge, persistent uncertainties, and a climate that will continue to change.

## 6 Conclusions

The effects of armed conflict and war are severe and long lasting for societies and, importantly, greatly increase their climate‐related vulnerability. Comprehensive and transdisciplinary efforts are needed to fully understand the multifaceted links between climate and conflict and appropriate responses. Associated questions at the frontier of current knowledge come into focus as societies increasingly respond to the changing climate, advance inclusive sustainable development, and support stable, peaceful states. Against a backdrop of finite resources and ongoing choices, it is important to understand the interactions between climate change and the risk of violent conflict, along with the synergies and trade‐offs among all the options for response.

## Conflict of Interest

The authors declare no financial or other conflicts of interests.
